# In Situ Thermography During Laser Powder Bed Fusion of a Nickel Superalloy 625 Artifact with Various Overhangs and Supports

**DOI:** 10.6028/jres.126.005

**Published:** 2021-03-12

**Authors:** Benjamin Molnar, Jarred C. Heigel, Eric Whitenton

**Affiliations:** 1 National Institute of Standards and Technology, Gaithersburg, MD 20899, USA; 2 Third Wave Systems, Eden Prairie, MN 55344, USA

**Keywords:** 3D build, additive manufacturing, IN625, model validation, nickel super alloy 625, overhangs, powder bed fusion, reference artifact, temperature measurement, thermography

**Data DOI:** https://doi.org/10.18434/M32112

## 1 Summary

This document provides details on the experiment and associated measurement files available for download in the dataset "*In Situ Thermography During Laser Powder Bed Fusion of a Nickel Superalloy 625 Artifact with Various Overhangs and Supports*." The measurements were acquired during the fabrication of a small nickel superalloy 625 (IN625) artifact using a commercial laser powder bed fusion (LPBF) system. The artifact consists of two half-arch features with increasing slopes for overhangs. These overhangs range from 5° from vertical to 85° from vertical in increments of 10°. The artifact geometry and process are controlled to ensure consistent processing along the overhang geometry. This control enables the effect of overhang geometry and support structures to be isolated from effects of inter-layer scan strategy variations. The measurements include high-speed thermography of each layer, from which radiance temperature, cooling rate, and melt pool length are calculated.

The objective of this experiment and data dissemination is twofold. The first objective is to provide exemplar data for the modeling community to ensure that their models are properly accounting for the effect of overhang geometries and support structures in thermal models. The second objective is to provide fundamental insight into how overhanging geometries impact the LPBF process for researchers and process designers.

## 2 Data Specifications

**Table:** 

**NIST Operating Unit(s)**	Engineering Laboratory
**Format**	There are several types of data formats included in this dataset. Please refer to Sec. 4 for a description of each type of data.
**Instruments**	An EOSint M270D^1^ laser powder bed fusion system was used to fabricate the overhang structures. An IRCameras model IRC 912 infrared camera was used to perform thermography of the scan tracks. Details are provided in Sec. 3.
**Spatial or Temporal Elements**	These measurements were performed on August 1, 2018
**Data Dictionary**	N/A
**Accessibility**	All datasets^2^ submitted to *Journal of Research of NIST* are publicly available.
**License**	https://www.nist.gov/director/licensing

1 Certain commercial equipment, instruments, or materials are identified in this paper in order to specify the experimental procedure adequately. Such identification is not intended to imply recommendation or endorsement by the National Institute of Standards and Technology, nor is it intended to imply that the materials or equipment identified are necessarily the best available for the purpose.

2 The National Institute of Standards and Technology (NIST) uses its best efforts to deliver a high-quality copy of the Database and to verify that the data contained therein have been selected on the basis of sound scientific judgment. However, NIST makes no warranties to that effect, and NIST shall not be liable for any damage that may result from errors or omissions in the Database.

## 3 Methods

**Fig. 1:**
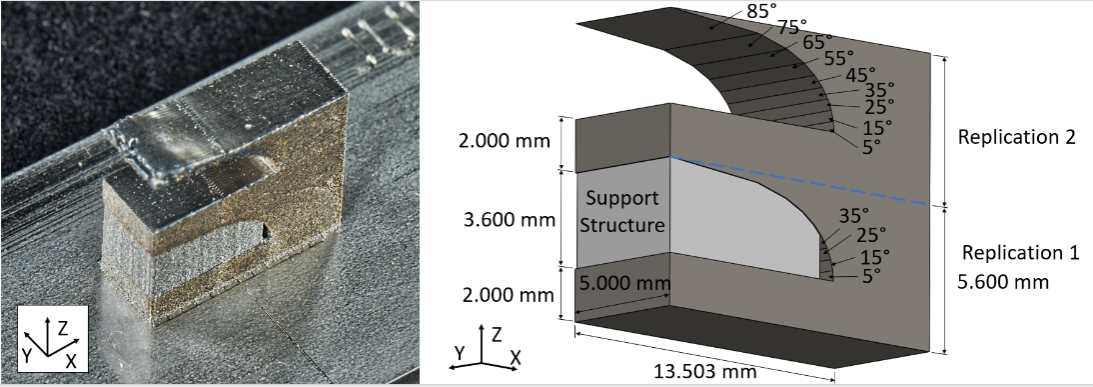
The part fabricated in this study. A) Picture of the completed part on the substrate. B) Illustration of the artifact with dimensions.

**Fig. 2:**
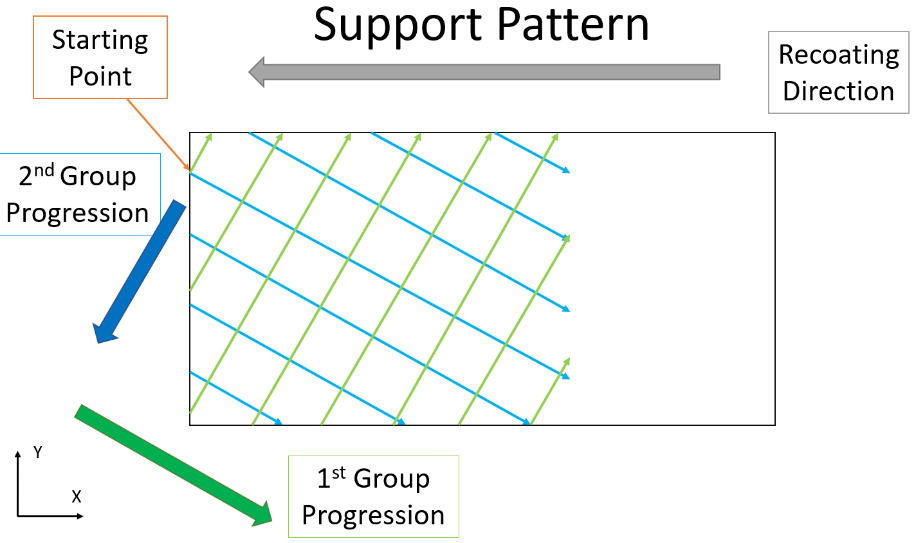
A simplified view of the support structure used in the study. In this study, supports are comprised of a 1 mm hatch pattern rotated 30° from the Y axis. The scans begin with the more vertical hatch lines (light green) and progress from upper left to lower right (in the direction of the dark green line). Following this, the more horizontal hatch lines (light blue) are scanned, progressing from upper right to lower left (in the direction of the dark blue line).

The experiment utilizes a commercial LPBF system to manufacture an IN625 artifact, depicted in Fig. 1. The artifact is designed to be 11.200 mm tall, 5.000 mm wide, and 13.503 mm long. It consists of two half-arches that are stacked on top of each other. These arches are the same geometry repeated twice, as indicated by the blue dashed line in Fig. 1B. The first (bottom) replication of the geometry includes a support structure beneath overhangs of 45° or greater, while the second (top) replication is built without the support structure. The support structure consists of a hatch pattern with 1 mm spacing, rotated clockwise 30° relative to the Y axis. This support structure is illustrated in Fig. 2.

Each arch is created on top of a rectangular base measuring 13.503 mm, 5.000 mm, and 2.000 mm in the X, Y, and Z axes respectively. The arches begin 5 mm from the right edge of the part and are built up using an increasingly significant overhang. The angle of the overhang ranges from 5° to 85°. The overhang angle increases by 10° every 20 layers (or 0.4 mm in the Z direction). The build strategy for this artifact has been controlled and is detailed in Sec. 3.3.

**Fig. 3:**
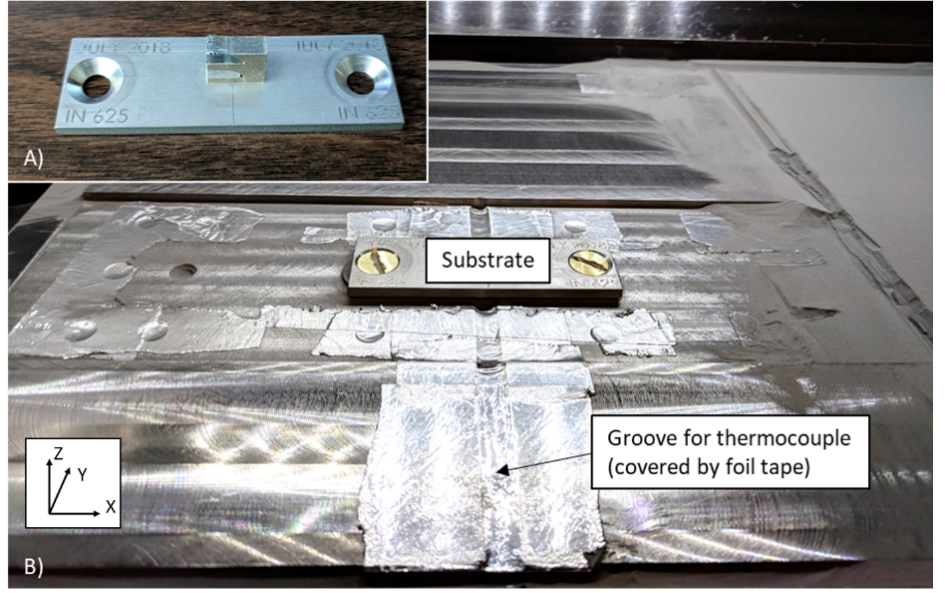
Depiction of the part on the substrate that is mounted to a larger build plate. A) the completed artifact on the substrate. B) the substrate mounted to the modified build plate.

The part was built on a small IN625 substrate, as shown in Fig. 3A. This substrate measures 75.0 mm long, 25.0 mm wide, and 3.2 mm tall. It has a countersunk hole on each side so that it can be bolted onto a larger steel build plate (250 mm square), as shown in Fig. 3B, that is mounted on the build platform of the LPBF system. The part is fabricated on a smaller substrate and not a full-size build plate to increase experiment throughput and to ease post-process testing by enabling the part to be removed without having to be cut off from a larger build plate. The substrate fits in a 3.0 mm deep recess in the steel build plate, which minimizes the amount of powder needed to be packed around the substrate for the build. There is a deeper groove in the steel build plate to accommodate a thermocouple to be welded to the bottom of the small substrate to track the temperature during a build. However, the substrate temperature was not measured during this experiment.

A high-speed infrared (IR) camera is used to measure the thermal history of each layer within the part geometry with the camera's region of interest (ROI) encompassing the entire build area. Measurement with an IR camera allows the thermal history to be measured and compared with the model predictions.

### 3.1 Powder

The powder used in this study was originally utilized in the 2018 Additive Manufacturing Benchmark Test Series (AM-Bench). For details on the original composition of the powder, please refer to [4]. This application is the third use of the powder, with the powder being sieved between uses according to manufacturer recommendations. No characterization of the powder in this state was made, nor were any powder samples collected.

### 3.2 Part Design

**Fig. 4:**
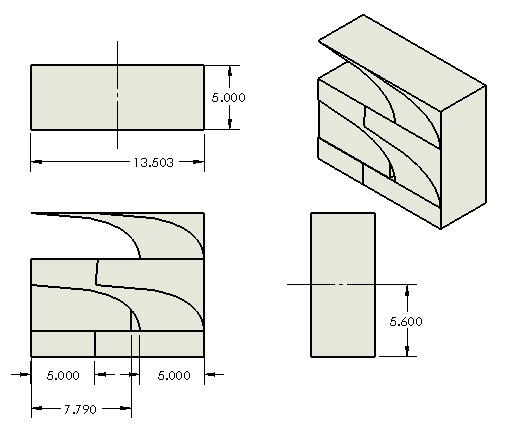
Engineering drawing of artifact. Units are in mm.

The artifact is presented in Fig. 4. The artifact is 13.503 mm long, 11.200 mm tall (5.600 mm for each repetition), and 5.000 mm wide. The part contains a prominent gradual overhang that spans the length of the part and gradually evolves from 5° to 85°, the most extreme overhang angle, by 10° increments every 20 layers (each 20 µm tall) or 0.4 mm.

**Fig. 5:**
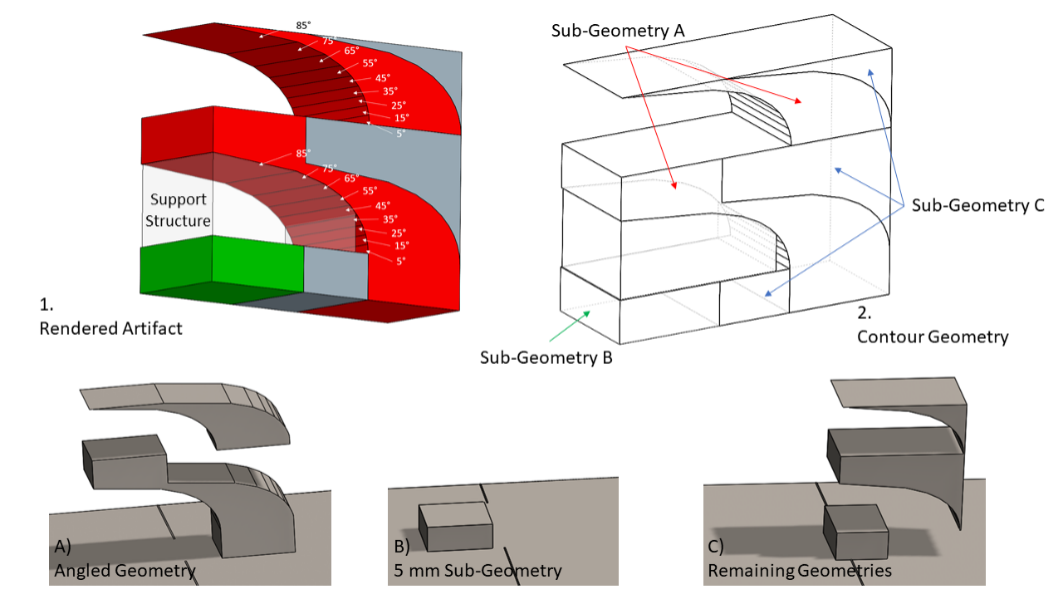
Illustrations of the artifact showing 1. A combined view of the artifact's sub-geometries 2. The contours of the artifact. Sub-geometries include A) The Angled Geometry at a constant 5 mm from the left edge after the first base B) The 5 mm square geometry on the first base C) The remaining geometries.

The experiment uses a constant scan strategy on a 5 mm area that follows the leftmost edge of the artifact once the angled layers begin. This sub-geometry is highlighted red in Fig. 5-1. The artifact has been divided into sub-geometries to allow the different parts of the build to be scanned separately. Stereolithography (STL) files of the part are available for download in the dataset under "CAD Files" to allow the part to be manufactured in subsequent studies using the same sub-geometries to control the scan strategy.

### 3.3 Build Strategy

This section presents the scan strategy and parameters used to execute the build. In each layer, the perimeter of the part is solidified using a contour scan.

The interior of the part cross-section is then solidified using skin and downskin scans. Skin is performed on areas directly over solidified material from the previous layer. Downskin solidifies areas with no, or minimal, solid material underneath. The scan path during the "skin" steps is a raster pattern aligned with either the X or Y axis, depending on whether the layer number is even (X-axis) or odd (Y-axis). The order in which internal geometries are scanned is dependent on which sub-geometry (A, B, and C in Fig. 5) the geometry belongs to. Scans occur in the order "Angled Geometry", then "5 mm Sub-Geometry", then "Remaining Geometries" or (A, then B, then C).

Finally, in layers that require it, the support structure is scanned (Fig. 2). Once the layer is complete the build plate is incremented down one layer height (20 µm) and a new layer of powder is spread across the build area. The following sub-sections provide details on each of these steps.

#### 3.3.1 Contour Scan Strategy

The contour of each feature on the part is scanned first using a programmed laser power of 100 W and a scan speed of 900 mm/s. For this part the beam offset is set to zero, meaning the center of the laser scan track traces the outline of the part. There is no information regarding the error of the laser path compared to the perimeter of the part in this study.

#### 3.3.2 Odd Layer Scan Strategy

**Fig. 6:**
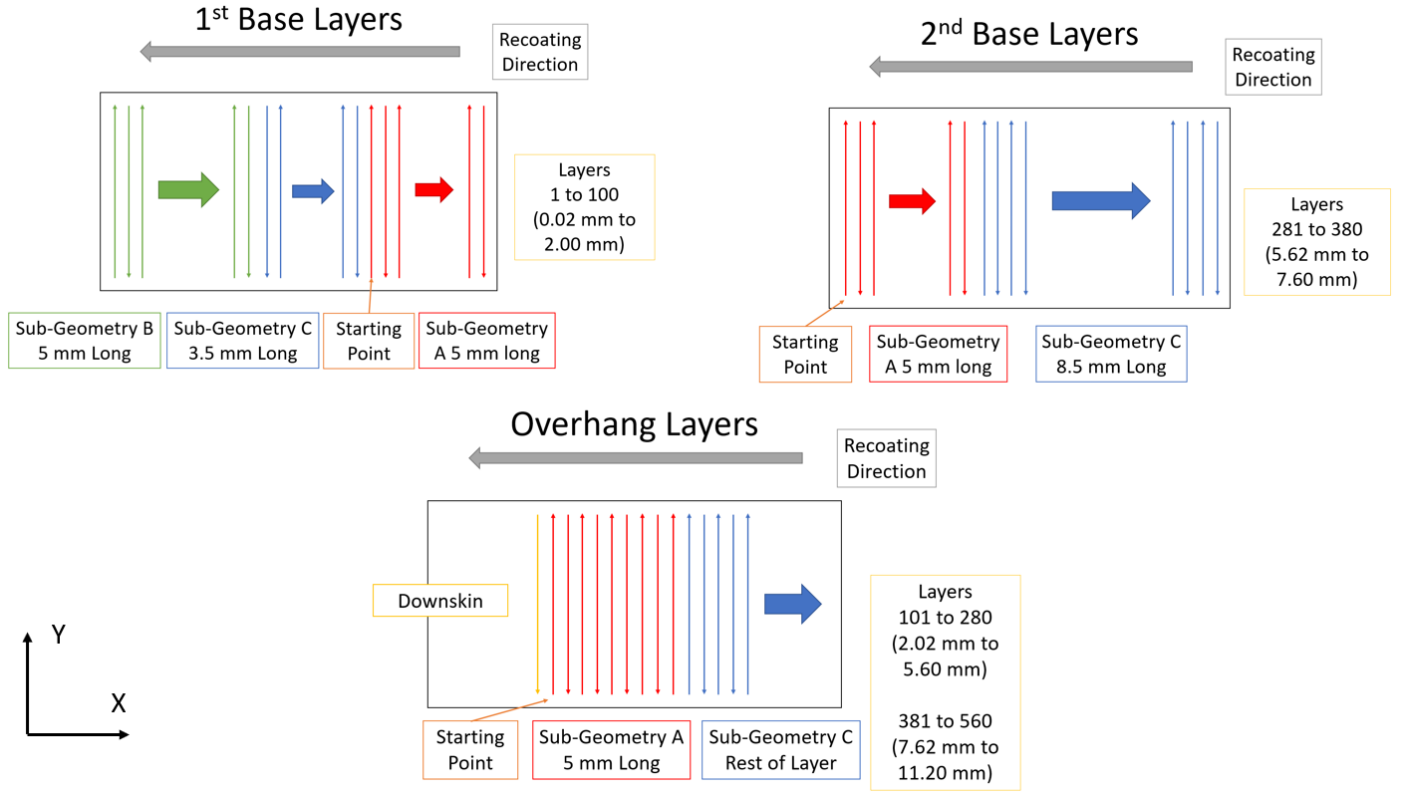
Scan strategy for odd numbered layers. For each layer Sub-Geometries A, B and C are scanned in order. Sub-Geometries A, B and C refer to directly refer to A, B and C in Fig. 5. In layers without Sub-Geometry B, the scan goes directly from A to C. Downskins occur directly after the skin portion of Sub-Geometry A.

Odd-numbered layers are scanned with the laser travelling at a speed of 800 mm/s with a programmed power of 195 W. Figure 6 illustrates the scan strategy for odd numbered layers. In odd layers, the laser scans in a raster pattern aligned with the Y axis. The first infill scan line begins in the lower left corner of Sub-Geometry A, and travels in the positive Y direction until the edge of the part is reached. After reaching the edge, the laser turns off, moves one hatch spacing in the positive X direction, and a new scan line is made in opposite direction (negative Y). The hatch spacing is the distance between two adjacent scan lines and is set to 0.1 mm in this study. This cycle is repeated until the layer completes. Note, the laser does not continue fully to the part's edge but starts and stops 0.03 mm from it.

The first base layers begin by scanning the rightmost 5 mm of the layer (Sub-Geometry A). After scanning the first sub-geometry, the laser starts at the left side and completes the layer (Sub-Geometries B and C). For the second base layers, the laser starts at the left side of the layer and progresses rightward for the duration of the layer (Sub-Geometries A and C). On angle layers, the laser pauses after the skin portion of Sub-Geometry A and performs a number of downskins on the far-left side, dependent on the overhang angle (specifically dependent on the amount of overhanging geometry in that layer). This downskin phenomenon is due to the machine's strategy for handling overhanging structures.

#### 3.3.3 Even Layer Scan Strategy

**Fig. 7:**
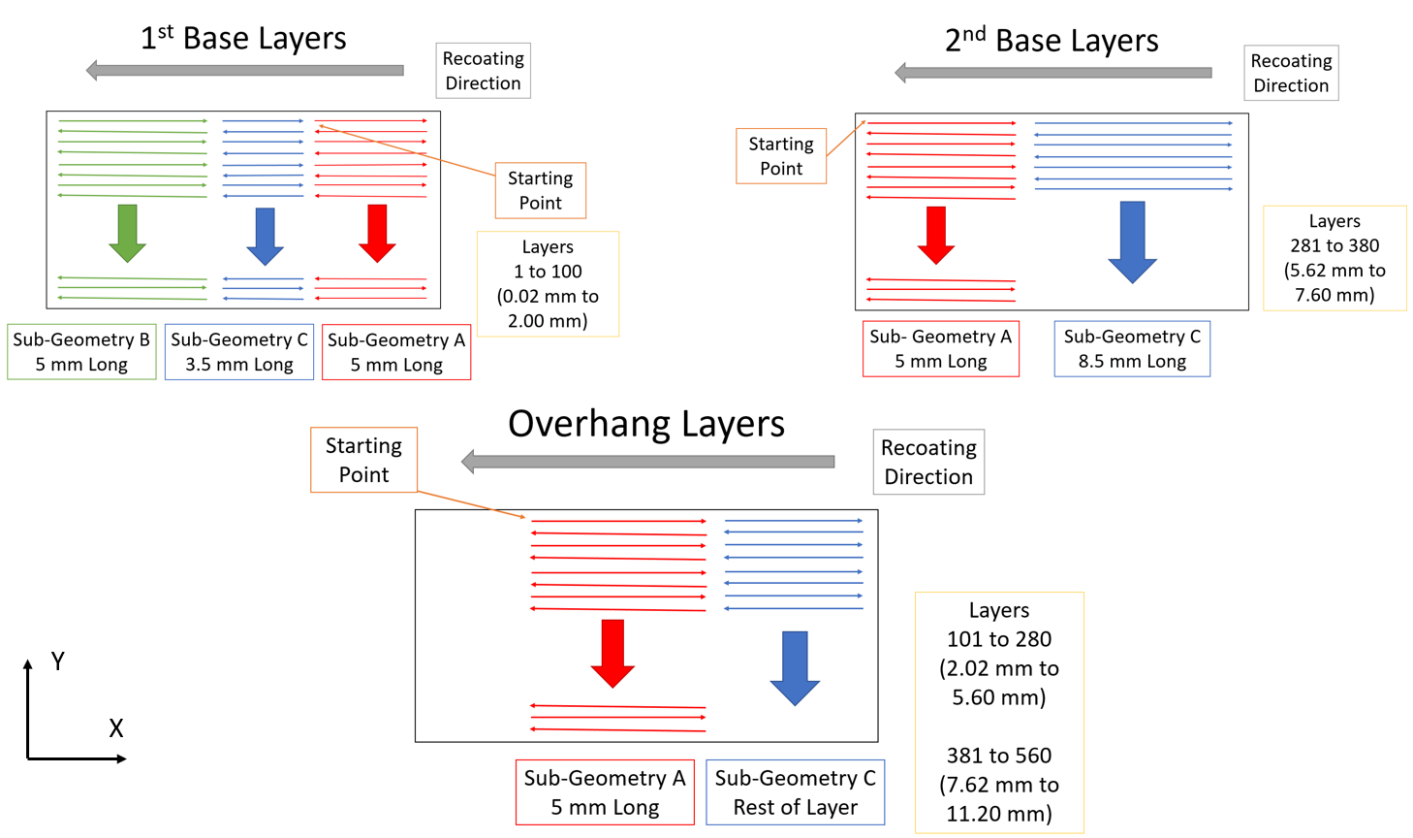
Scan strategy for even numbered layers. For each layer Sub-Geometries A, B and C are scanned in order. Sub-Geometries A, B and C refer to directly refer to A, B and C in Fig. 5. In layers without Sub-Geometry B, the scan goes directly from A to C.

All even numbered layers are processed by the laser travelling at a programmed speed of 800 mm/s and using a power of 195 W. Figure 7 illustrates the scan strategy for even numbered layers. In even layers the laser scans back-and-forth along the X axis. The first infill scan line begins in the upper left corner of Sub-Geometry A and scans rightward.

The first base layer begins by scanning the rightmost 5 mm of the part (Sub-Geometry A), scanning a raster pattern in this area until the sub-geometry is complete. Next the laser scans the leftmost 5 mm of the part (Sub-Geometry B), progressing downward until the sub-geometry is complete. Finally, the middle

3.5 mm long area is scanned (Sub-Geometry C), completing the layer. On the second base layer the laser begins by scanning the leftmost 5 mm sub-geometry of the layer (Sub-Geometry A). After this sub-geometry is completed the laser begins scanning the rest of the layer (Sub-Geometry C). For angle layers the leftmost 5 mm is scanned first (Sub-Geometry A). After this sub-geometry is finished, the laser moves onto the rest of the part, scanning the remainder as a continuous sub-geometry regardless of its size (Sub-Geometry C).

#### 3.3.4 Recoating

Recoating is performed using a solid high-speed steel (HSS) recoating blade, the recoating blade type specified for IN625. The recoating blade spreads powder across the powder bed surface at a speed of 80 mm/s.

### 3.4 Temperature Measurement

The in-situ temperature measurement system has been described at length in other publications [1, 2, 4]. However, a summary is provided here for reference. The camera is an IRCamera model IRC 912. A band-pass filter is used to limit the detectable wavelength range from 1350 nm to 1600 nm for a variety of reasons that are detailed in [2]. The integration time is 40 µs and the frame rate is 1800 frames per second. Each image frame is comprised of 360 horizontal pixels and 126 vertical pixels, which equates to a field of view of 12.06 mm wide (X axis) by 6.27 mm tall (Y axis). The instantaneous field of view (iFOV) of each pixel is 33.5 µm wide and 49.8 µm tall. The asymmetric iFOV is a result of the camera's angled view at the build plane, as the camera's line of sight makes a 41° angle with the horizontal build plane.

For each pixel, the camera measures a signal that is related to temperature via the following equation:

$S_{\text{meas}}=ɛF\left(T_{\text{bb}}\right)=F(T_{\text{rad}})$ (1)^3^ 3It should be noted that 𝑆_meas_ is approximated by this equation and the measured camera signal does not necessarily equal the given functions of radiant temperature. 


where $S_{\text{meas}}$ is the camera signal in digital levels (DL), $T_{\text{bb}}$ is the blackbody temperature in K, $T_{\text{rad}}$ is the apparent radiance temperature of the graybody additively manufactured part (also called $T_{\text{app}}$ in some publications), and ɛ is the effective emissivity of the target surface of the object [2]. Effective emissivity is a dimensionless value between 0 and 1. Only for perfectly emitting blackbodies does ɛ= 1, all other bodies emit a fraction of the blackbody radiation. Consequently, the camera measures a signal in response to this radiance temperature, $T_{\text{rad}}$ in K, and the true temperature of the object can be calculated only if ɛ is known. The function relating $T_{\text{rad}}$ to $S_{\text{meas}}$ is defined by the Sakuma-Hattori equation [5] and its inverse:

$F\left(T_{\text{rad}}\right)=S_{\text{meas}}=\frac{C}{\exp\left(\frac{c_{2}}{AT_{\text{rad}}+B}\right)-1}$ (2)

F-1S=Trad=c2AlnCS+1-BA (3)

where ${\text{c}}_{2}$ is the second radiation constant (0.014388 m · K) and the coefficients *A*, *B*, and *C* are determined via the blackbody calibration procedure outlined by Lane and Whitenton [2]. A calibration blackbody is first used to create a two-point non-uniformity correction (NUC), then a series of measurements are performed with the calibration blackbody incrementally set to a range of temperatures covering the detectable range of the camera (550 °C to nearly 1100 °C), which is a function of the camera settings and optical system. The coefficients *A* = 2.6650, *B* = -800.70, and *C* = 1.9400 × 10^6^ are determined through the blackbody calibration reported in Ref. [4].

### 3.5 Measurement Uncertainty

The three measurands calculated from thermography results and provided in the data files include radiance temperature, melt pool length, and cooling rate. While users can re-calculate melt pool length, cooling rate, or true temperature themselves based on the radiance temperature data and a derived or assumed emissivity, this section provides measurement uncertainty values under similar conditions and assumptions made in Heigel *et al*. [6]. These conditions include the following: 1) true temperature is calculated using an assumed emissivity of ɛ = 0.221 (determined from Lane *et al*. based on observed solidification during single track measurements on IN625 without powder [7]) 2) melt pool length and cooling rates are calculated based on this $T_{true}$ 3) Cooling rate is determined from the temperature range $T_{true}$ = 1290 °C to 1000 °C.

The combined uncertainties, u_c_, for these measurands under these conditions are as follows, and provided as 68% confidence interval (or ±1σ assuming normal probability distribution) [8]. Combined uncertainty for true temperature, $u_{c}(T_{true})$ = 8.1%, in terms of units [K/K]. This incorporates combined uncertainty for emissivity u_c_(∈) = 0.036. Combined uncertainty for melt pool length is $u_{c}(L)$ = 125 µm, and cooling rate $u_{c}(\overset{\cdot }{T})$ = 0.5×10^5^ [°C/s].

## 4 Data Files

The dataset consists of 89 compressed zip files and two MATLAB functions. Each compressed file contains thermal videos and MATLAB data structures with the measurement data for either five or ten layers. These zip files contain the thermal videos and measurement data for all 560 layers of the part during the build. The name of each compressed file briefly describes the study, the layers, and the features being manufactured in those layers.

### 4.1 Thermal Video Descriptions

**Fig. 8:**
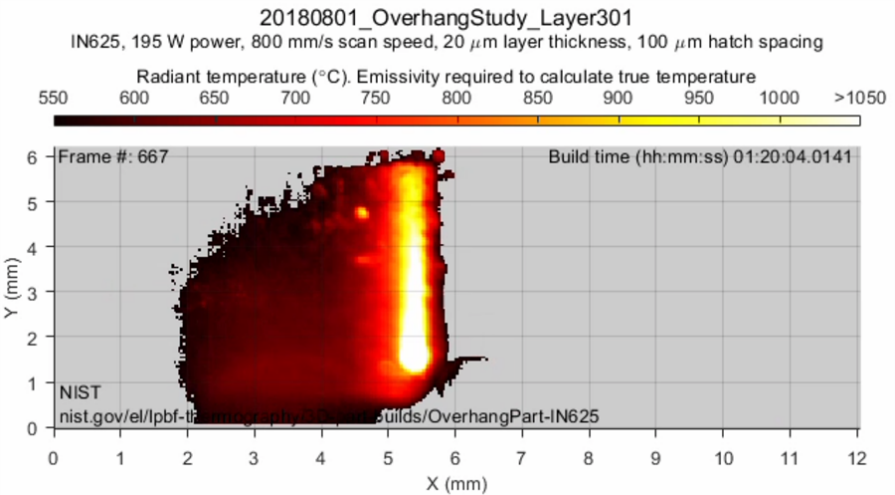
Example thermal video frame showing a part of Layer 301's thermal history. These videos provide quick references for data verification purposes.

Each layer's data file (.mat) is accompanied by a .mp4 thermal video showing the build process of the layer. A frame from such a thermal video is shown in Fig. 8. The thermal videos allow for a preview of the build process of each layer, which can then be cross referenced against the data file for that layer. The title at the top of each video frame describes the data file the thermal video corresponds to. Below the title, several important parameters are given such as the material, scan speed, layer thickness, and hatch spacing. A color bar defines the radiant temperature associated with each color. Since emissivity must be known to calculate true temperature, these videos only display radiant temperature. A process for calculating true temperature is described in Sec. 4.3.

The grid below the color displays the measured radiant temperature on that frame. Frame number, which starts at 1 for every layer and increments with successive frames, can be found in the top left. Build time, which denotes the time since the beginning of the build, can be found in the top right. A NIST logo and website link for this dataset is shown in the bottom left. The X and Y axes of the grid correspond to the X and Y axes of the part, where the origin is defined by camera placement. The units for these axes are millimeters.

In the example frame shown in Fig. 8 the laser is travelling downward in the negative Y direction. This can be deduced from the position of the laser head and the trailing melt pool, which shows a gradually decreasing temperature as distance from the laser head increases. The example frame in Fig. 8 also shows that the laser has scanned a region on the left (negative X) and is travelling rightward. The preliminary observations made regarding Fig. 8 can be verified with the predicted scan pattern in Fig. 6 and the data file associated with Layer 301.

A more detailed description of the thermal videos included in this dataset is described by Heigel *et al*. [4].

### 4.2 MATLAB Data Structure Descriptions

**Fig. 9:**
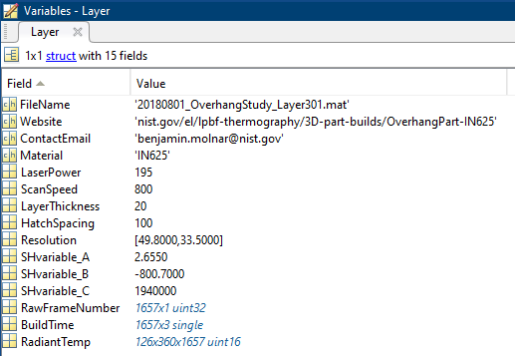
Example data file for Layer 301. Identifying information for the layer is given as well as contact information, build parameters, camera variables and calibration, the recorded radiant temperature in degrees Celsius, frame number and build time.

A data file is associated with each layer to give the measured radiant temperature, time, frame number, and a variety of other variables associated with the build process. Each data file is in .mat format and is named for the layer it describes. An example data file for Layer 301 is shown in Fig. 9.

Each layer data file is a 1x1 structure containing 15 fields. The first field, FileName, describes the name of the .mat file. Website gives a url to this dataset. ContactEmail contains an email that can be contacted to address any questions or concerns regarding the dataset. Material describes the material used in the study, in this case IN625. LaserPower describes the laser power in W. ScanSpeed gives the laser scan speed in mm/s. LayerThickness is the thickness of each layer in microns. HatchSpacing refers to the hatch spacing used in the build in microns. Resolution refers to the camera's iFOV in the Y and X directions respectively. These values are in units of µm /pixel. SHvariable_A, SHvariable_B, and SHvariable_C are predetermined values which refer to the variables A, B, C in Eqs. (2) and (3), which can be used to calculate true temperature. The values for these variables are determined by blackbody calibration. RawFrameNumber gives a list of the raw frame numbers recorded for each layer. BuildTime gives a list of the hours, minutes, and seconds since the start of the build for each recorded frame in the layer. Hours, minutes, and seconds are in separate columns. RadiantTemp reports the measured radiant temperature for every pixel in each recorded frame. This field is three dimensional due to the X and Y dimensions of the frame, and the stacking of each frame in the time dimension.

A more detailed description of the data files included in this dataset is described by Heigel *et al*. [4].

### 4.3 Description of the MATLAB Functions

There are two MATLAB functions provided. "MakeRadiantTempThermalVideo.m" will recreate the thermal videos provided in the dataset. The input of the function is the "Layer" data structure contained in each MATLAB data file. Stepping through the function should help a user to understand how the data in the structure is used. The second function is called "ConvertToTrueTemp.m" and can be used to convert the radiance temperature measurements that are provided in the "Layer" structure into thermodynamic temperature.

The function "ConvertToTrueTemp.m" requires two inputs: the "Layer" MATLAB structure and an assumed emissivity correction factor. It is the responsibility of the user to assume an effective emissivity. In this function, the radiance temperature is first converted back to $S_{\text{meas}}$ using Eq. (2) and the values of *A*, *B*, and *C* provided in the "Layer" structure. Then the thermodynamic temperature is calculated using Eqs. (1) and (3) and the assumed effective emissivity.

## 5 Impact

The purpose of this dataset is for the comparison of measured temperatures to that of predictions by process models that simulate the fabrication of the overhang structure; however, care must be taken because emissivity of the surface during processing must be known to calculate the thermodynamic temperature of each layer. Since the microstructure, strain, and distortion are a direct result of the thermal history the material experiences during the process, accurate models of those phenomena likely depend on accurate, validated, thermal process models. In addition to the above-mentioned comparison, the measurements of the thermal history can be used by material scientists to understand the phenomena observed in the observed microstructure.
